# Supplementary material for: Complexome profiling of the Chlamydomonas psb28 mutant reveals TEF5 as an early PSII assembly factor
Source: Plant Cell. 2025 Mar 17;37(6):koaf055. doi: 10.1093/plcell/koaf055 (PMC12164590; doi:10.1093/plcell/koaf055)
Supplement: koaf055_Supplementary_Data [file koaf055_supplementary_data.zip › psb28_interactive.zip!/plots/intensity/qualityPlots/peptides/ATP5_Cre16.g680000.t1.2.html]

### Cre16.g680000.t1.2

Trivial name: ATP5  
SeqCoverage [median]: 35.60  
Mass: 25.88 kDa  
  
 

| Sequence | MissedCleavage | Mass [Da] | wtIntensity | muIntensity | Score |
| --- | --- | --- | --- | --- | --- |
| DMFATK | 0 | 711.33 | 11986.09 | 19479.96 | 124.89 |
| DMFATKDVTEVTKR | 2 | 1639.82 | 741.33 | 0.00 | 40.94 |
| DVTEVTK | 0 | 790.41 | 11574.82 | 15469.26 | 147.34 |
| DVTEVTKR | 1 | 946.51 | 50175.72 | 60603.61 | 186.13 |
| KLEEFNNLVTK | 1 | 1333.72 | 23945.90 | 37536.70 | 190.53 |
| LATDPFLPDAFR | 0 | 1361.70 | 9627.17 | 33002.57 | 141.88 |
| LATDPFLPDAFRR | 1 | 1517.80 | 54340.81 | 109627.15 | 149.23 |
| LENDLK | 0 | 730.39 | 27589.88 | 38765.84 | 128.38 |
| LVDMSQAK | 0 | 890.45 | 58440.69 | 72911.73 | 180.93 |
| QAQAFVDPGFK | 0 | 1206.60 | 46981.86 | 73569.16 | 185.29 |
| RLATDPFLPDAFR | 1 | 1517.80 | 6225.58 | 0.00 | 59.61 |
| RLATDPFLPDAFRR | 2 | 1673.90 | 4387.05 | 1754.64 | 68.97 |
| SHPEIR | 0 | 737.38 | 12862.91 | 10519.01 | 118.06 |
| VQDELYQLVEVFK | 0 | 1608.84 | 9534.68 | 19370.91 | 257.19 |
|  |  |  | 328414.5 | 492610.6 |  |
